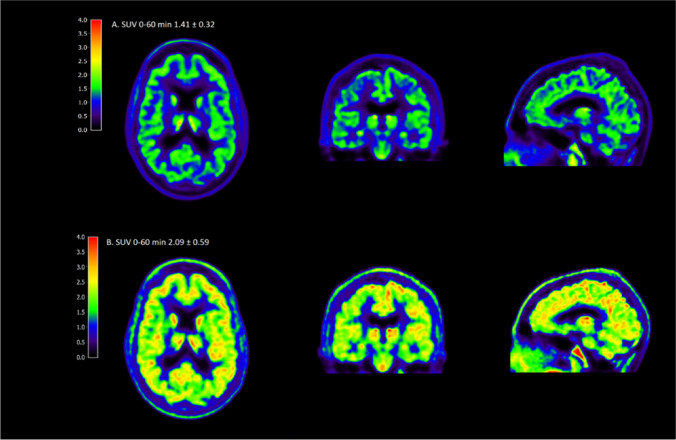# Evaluation of P-glycoprotein function at the blood–brain barrier using [^18^F]MC225-PET

**DOI:** 10.1007/s00259-021-05419-8

**Published:** 2021-06-05

**Authors:** Pascalle Mossel, Lara Garcia Varela, Wejdan M. Arif, Chris W. J. van der Weijden, Hendrikus H. Boersma, Antoon T. M. Willemsen, Ronald Boellaard, Philip H. Elsinga, Ronald J. H. Borra, Nicola A. Colabufo, Jun Toyohara, Peter Paul de Deyn, Rudi A. J. O. Dierckx, Adriaan A. Lammertsma, Anna L. Bartels, Gert Luurtsema

**Affiliations:** 1 Department of Nuclear Medicine and Molecular Imaging, University of Groningen, University Medical Center Groningen, Hanzeplein 1, 9713 GZ Groningen, The Netherlands; 2 College of Applied Medical Science, Department of Radiological Sciences, King Saud University, Riyadh, Saudi Arabia; 3 Department of Radiology & Nuclear Medicine, Amsterdam UMC, Vrije Universiteit Amsterdam, de Boelelaan 1117, Amsterdam, The Netherlands; 4 Department of Pharmacy-Drug Sciences, University of Bari Aldo Moro, Bari, Italy; 5 Research Team for Neuroimaging, Tokyo Metropolitan Institute of Gerontology, 35-2 Sakaecho, Itabashiku, Tokyo, 1730015 Japan; 6 Department of Biomedical Sciences, Neurochemistry and Behaviour, Institute Born-Bunge (IBB), University of Antwerp, Wilrijk, Antwerp, Belgium; 7 Department of Neurology, Alzheimer Center Groningen, University Medical Center Groningen (UMCG) and University of Groningen, Hanzeplein 1 9713, GZ Groningen, The Netherlands; 8 Faculty of Medicine and Health Sciences, University of Antwerp, Wilrijk, Antwerp, Belgium

P-glycoprotein (P-gp) is an ATP-dependent efflux transporter located at the blood–brain barrier (BBB), involved in the transport of a variety of neurotoxic substances out of the brain. Alterations in P-gp function play an essential role in the pathophysiological mechanisms underlying neurodegenerative disorders. The most widely used tracer to measure BBB P-gp function in vivo is *(R)*-[^11^C]verapamil [1]. However, *(R)-*[^11^C]verapamil is an avid P-gp substrate, and its low uptake hampers the measurement of increases in P-gp function. In order to overcome this limitation, [^18^F]MC225 was developed as a novel PET tracer to measure P-gp function in vivo. [^18^F]MC225 is a weaker P-gp substrate and has shown higher brain uptake than *(R)-*[^11^C]verapamil at baseline in preclinical studies [2]. This may facilitate the evaluation of both increases and decreases in P-gp function. In addition, the longer half-life of fluorine-18 enables the use of [^18^F]MC225 in centers without an onsite cyclotron.

These standardized uptake value (SUV) images show one of the first [^18^F]MC225 PET brain scans in a healthy human subject in both unblocked (A) and blocked (B) P-gp state. Blocking was achieved by continuous intravenous administration of the specific P-gp inhibitor cyclosporin (2.5 mg/kg/h), starting 30 min prior to the scan. Quantitatively, the whole brain grey matter volume of distribution V_T_ changed from V_T_ = 4.38 at baseline to V_T_ = 5.48 after cyclosporin administration, showing higher uptake at baseline levels compared with previously described data of [^11^C]verapamil (V_T_ = 1.28 at baseline, V_T_ = 2.00 after P-gp inhibition) [3], illustrating [^18^F]MC225 as a promising tracer to measure BBB P-gp function in humans.